# Safety and efficacy of fixed-dose combination of dapagliflozin and saxagliptin in patients with type 2 diabetes mellitus – a phase 4 study in India

**DOI:** 10.3389/fendo.2025.1528801

**Published:** 2025-03-03

**Authors:** S. K. Wangnoo, Sanjay Kumar Bhadada, Faraz Farishta, Girithara Gopalakrishnan Jayaram Naidu, Indira Pattnaik, K. N. Manohar, K. P. Singh, Sandeep Kumar Gupta, H. S. Bharath, Sujoy Ghosh

**Affiliations:** ^1^ Apollo Centre for Obesity, Diabetes & Endocrinology of Indraprastha Apollo Hospitals, New Delhi, India; ^2^ Department of Endocrinology, Post Graduate Institute of Medical Education and Research, Chandigarh, India; ^3^ FS Endocrine and Diabetes Centre, Hyderabad, India; ^4^ Department of Diabetology, KG Hospital Coimbatore, Coimbatore, Tamil Nadu, India; ^5^ Department of Medicine, Sparsh Hospitals and Critical Care (P) Ltd, Bhubaneswar, Odisha, India; ^6^ Department of Internal Medicine, Manipal Hospital, Bangalore, Karnataka, India; ^7^ Department of Endocrinology, Fortis Hospital, Mohali, Punjab, India; ^8^ Department of Medicine, M V Hospital and Research Centre, Lucknow, Uttar Pradesh, India; ^9^ Department of Medical Affairs, AstraZeneca Pharma India Ltd, Bangalore, Karnataka, India; ^10^ Department of Endocrinology, IPGME & R and SSKM Hospital, Kolkata, India

**Keywords:** prospective, single-arm, dapagliflozin, saxagliptin, fixed-dose combination, diabetes mellitus type II, India, safety

## Abstract

**Objective:**

To determine the post-marketing safety profile of a once-daily fixed-dose combination (FDC) of dapagliflozin (10 mg) and saxagliptin (5 mg) given orally for 24 weeks or until discontinuation, in Indian patients with type 2 diabetes mellitus (T2DM) who are on stable dose of metformin.

**Design:**

Prospective, single-arm, multicenter study

**Setting:**

Adult patients with T2DM enrolled from April 2021 to March 2023 across 9 study sites in India

**Outcome measures:**

The primary objective was to determine the adverse event (AE) profile of the FDC. Additionally, we assessed changes in glycated hemoglobin (HbA1c), fasting plasma glucose (FPG), systolic blood pressure, and body weight at 24 weeks, compared to baseline.

**Results:**

Of the 196 patients (median age [range]: 53 [20 to 78] years) analyzed, 61.2% were males with mean ± standard deviation [SD] duration of T2DM of 7.1 ± 5.7 years. Overall, 111 (56.6%) presented with ≥1 comorbidity; the most frequent being hypertension (57; 29.1%). At 24 weeks, a total of 22 patients (11.2%) experienced 40 AEs; the majority of them had mild AEs. The most frequent AEs included urinary tract infection (5; 2.6%), pyrexia (5; 2.6%), nasopharyngitis (3; 1.5%), and balanoposthitis (3; 1.5%). The AEs of special interest reported were genital tract infection (3; 1.5%) and hypoglycemia (1; 0.5%). No serious AEs were reported. None of the AEs required treatment discontinuation. Three (1.5%) patients had AEs leading to temporary interruption of the study drug. No deaths were reported in this study. The mean absolute change in HbA1c (1.2% ± 1.1%), FPG (24.4 ± 62.9 mg/dL), and weight (2.1 ± 4.0 kg) from baseline to 24 weeks was statistically significant (p < 0.0001).

**Conclusion:**

Our study demonstrated the safety and efficacy of once-daily FDC of dapagliflozin and saxagliptin when added to metformin in Indian patients with T2DM.

## Introduction

The incidence of diabetes has consistently shown an upward trend, particularly in Southeast Asian nations, with India ranking second to China as per the disease burden (1–3). According to the International Diabetic Federation Diabetic Atlas (2021), about 74.2 million people in India have diabetes mellitus, majorly type 2 diabetes mellitus (T2DM), and this number is expected to reach 125 million by 2045 (3). Despite therapeutic interventions, more than two-thirds of patients have poor glycemic control (glycated hemoglobin [HbA1c] ≥7%) (4, 5).

The Asian-Indian phenotype of T2DM presents a unique cardiometabolic risk profile, necessitating a multifaceted therapeutic approach for optimal glycemic control and prevention of cardiovascular complications (6). As a result, the rational combination of different therapeutic options is crucial in prioritizing patient-centric management. The American Diabetes Association recommends initial combination therapy for patients with HbA1c levels 1.5% to 2.0% above the target (7). Sodium-glucose cotransporter-2 inhibitors (SGLT2i) and dipeptidyl peptidase 4 inhibitors (DPP4i) are preferred options due to their superior efficacy in glycemic control along with reduction in cardiovascular risk and an acceptable safety profile, particularly in the context of Indian diabetic setting (8).

Numerous studies have demonstrated that the combination of dapagliflozin (an SGLT2i) and saxagliptin (a DPP4i) with metformin resulted in better glycemic control along with the reduction in body weight and blood pressure compared to individual agents, with an acceptable safety profile (9–13). Available evidence demonstrated comparable safety and tolerability of dapagliflozin and saxagliptin combination to individual agents added to metformin (13, 14). Additionally, the incidence of urinary and genital tract infections was lower with the combination regimen, compared to the sequential add-on regimen (13).

Recent trends in prescription pattern practices indicate an increased preference for fixed-dose combinations (FDCs) owing to their simplified dosing schedule and reduced pill burden, contributing to enhanced treatment compliance (15). A once-daily FDC of dapagliflozin and saxagliptin was approved for the management of T2DM by the European Medicines Agency in 2016 (16) and the Food and Drug Administration in 2017 (17). It received marketing approval from the Drug Controller General of India in 2019 (18). This FDC is indicated as an adjunct to diet and exercise to improve glycemic control in adults with T2DM who have inadequate glycemic control with metformin and/or sulfonylurea and either of the individual components of the combination or who already received treatment with dapagliflozin and saxagliptin (16). Considering limited data on the safety of this FDC in the Indian population, the present study was conducted in response to the mandate from the Heath Authority of India to determine the post-marketing safety profile of QTERN^®^ (FDC of dapagliflozin 10 mg and saxagliptin 5 mg) among Indian patients with T2DM.

## Methods

### Study design

This was a prospective, single-arm, multicenter study conducted at 9 study sites across India between April 2021 and March 2023. The study protocol (NCT04445714) was approved by the Independent Ethics Committees/Institutional Review Boards of all the participating centers. The study was conducted in accordance with the Declaration of Helsinki, the International Council for Harmonisation, good clinical practices, good pharmacoepidemiology practices (GPP), and the applicable legislations. Written informed consent was obtained from all the patients before enrollment. The reporting has been done following the Strengthening the Reporting of Observational Studies in Epidemiology (STROBE) checklist for cohort studies (19).

### Study population

The study included patients aged ≥18 years with T2DM (HbA1c ranging between >7% and ≤10%) on a stable dose of anti-diabetic medications for ≥3 months before enrollment. Patients with type 1 diabetes mellitus, prior treatment with an SGLT2i, glucagon-like peptide-1 agonist, or DPP4i, severe hepatic impairment, moderate to severe renal impairment, cardiovascular disease within 3 months prior to enrollment, and severe uncontrolled hypertension were excluded. Detailed inclusion and exclusion criteria are presented in **Supplementary Table S1**.

Enrolled patients were administered once-daily FDC of dapagliflozin 10 mg and saxagliptin 5 mg orally at the same time of a day for 24 weeks or until discontinuation of the study drug due to either adverse events (AEs) or major and/or frequent hypoglycemic events, or other reasons such as consent withdrawal by the patient, whichever occurred earlier. All the patients were on stable dose of metformin (ranging between 1000 and 2000 mg) at enrollment. Patients were allowed to receive additional glucose-lowering drugs as per investigator’s discretion. Concomitant use of weight-loss medications, antiviral drugs, and systemic glucocorticoids was prohibited.

### Study objectives and data collection

The primary objective was to determine the safety of dapagliflozin and saxagliptin FDC at 24 weeks by evaluating the incidence of AEs, serious AEs, AEs leading to discontinuation, AEs of special interest (AESIs) including major hypoglycemic events, urinary and genital tract infections, volume depletion, diabetic ketoacidosis, fractures, renal events, and hospitalization for heart failure (HHF). The secondary objective included efficacy parameters at 24 weeks, assessed by the changes in HbA1c, fasting plasma glucose (FPG), systolic blood pressure (SBP), and body weight at 24 weeks compared to baseline. At baseline, data were collected for demographics, medical history, HbA1c, FPG, vital signs, physical examination, electrocardiogram (ECG), and laboratory parameters and entered into electronic case report forms. Additionally, data regarding concomitant glucose-lowering medications were also recorded at baseline. Subsequently, patients underwent follow-up assessments at Weeks 8, 16, and 24 (**Supplementary Figure S1**).

### Statistical analysis

A sample size of 200 patients was deemed to be adequate to estimate AEs associated with the FDC at the incidence rate of 8%, with a margin of error of 4% and overall incidence rate of AEs at 50%, with a margin of error of 7%. The safety population included all enrolled patients who had signed the informed consent form and received at least one dose of the FDC. All reported AEs were included in the analysis.

Statistical analyses were performed using SAS (version 9.4 or higher) software (SAS Institute Inc USA). Data were summarized using descriptive statistics. Continuous variables were summarized using the number of observations, mean, standard deviation (SD), median, and range as appropriate. Categorical values were summarized using the number of observations and percentages. The mean change in HbA1c from baseline to 24 weeks was analyzed using a paired t-test/Wilcoxon signed-rank test at a 5% level of significance. The last treatment observation was carried forward to impute the missing data at 24 weeks. A p-value < 0.05 was considered statistically significant.

## Results

**Figure 1:**
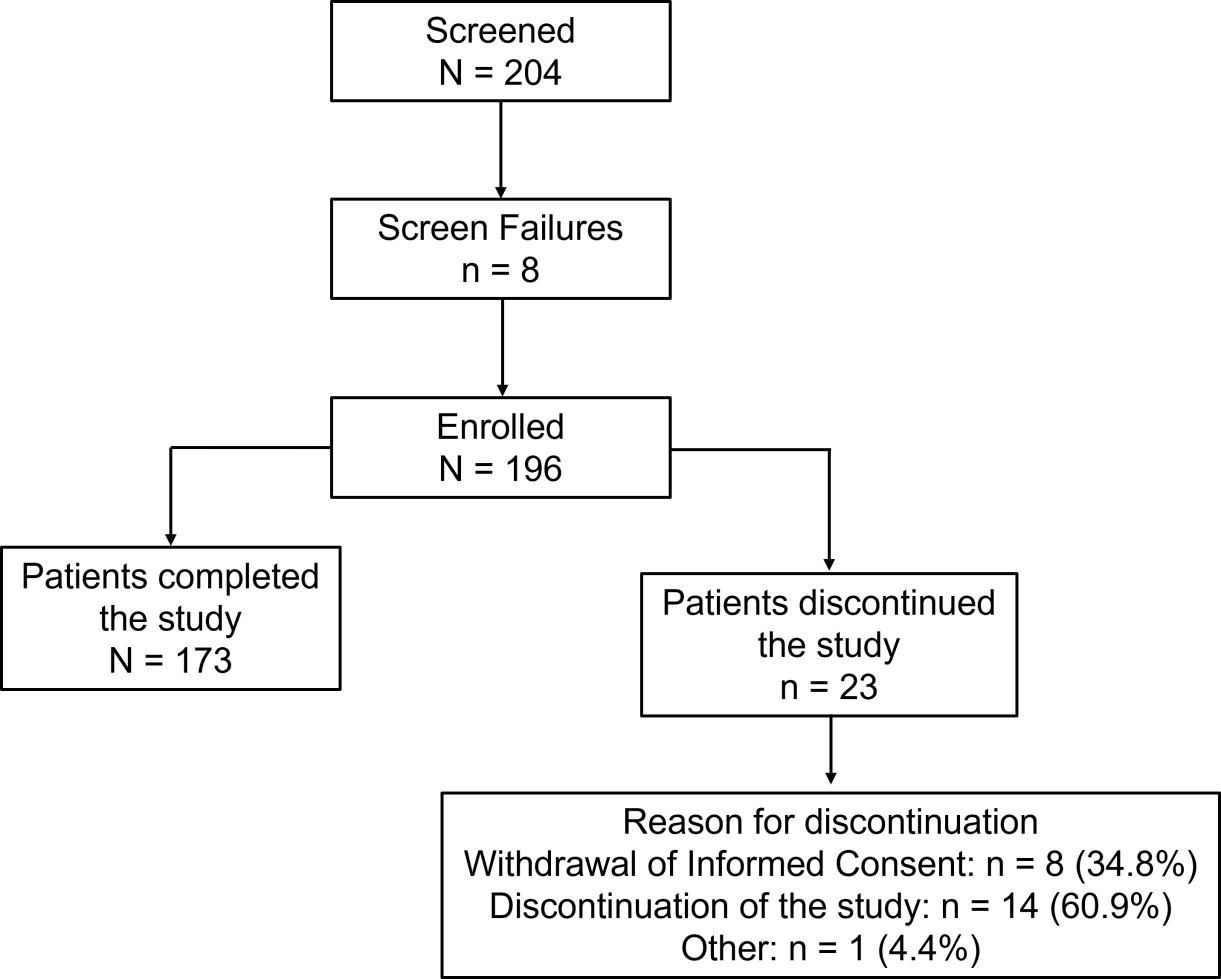
Patient disposition of the study. Screened patients are those who signed the informed consent. Percentages for the ‘reasons for discontinuation’ are based on the number of patients who discontinued the study.

Of the 204 patients screened, 196 patients were enrolled in the study; 173 (88.3%) patients completed the study, and 23 (11.7%) patients discontinued the study. The patient disposition along with reasons for discontinuation is presented in **Figure 1**. The median age of patients was 53 years (range: 20 to 78 years); the majority were males (120; 61.2%) with a mean ± SD body weight of 74.2 ± 12.6 kg and mean ± SD body mass index of 28.0 ± 4.4 kg/m^2^. The mean ± SD duration of T2DM was 7.1 ± 5.7 years and the mean ± SD baseline HbA1c was 8.6% ± 0.8%. Overall, 111 patients (56.6%) presented with ≥1 comorbidity. The most frequent comorbidities were hypertension (57; 29.1%) and dyslipidemia (36; 18.4%) (**Table 1**). Commonly prescribed glucose-lowering medications in addition to background metformin included glimepiride, glibenclamide, and gliclazide.

**Table 1:** Baseline characteristics.

Characteristics	Safety Population N = 196, n (%)
Age (years), median (range)	53.0 (20.0, 78.0)
Male, n (%)	120 (61.2)
BMI (kg/m^2^)^*^	28.0 ± 4.4
Weight (kg)^*^	74.2 ± 12.6
HbA1c (%)^*^	8.6 ± 0.8
FPG (mg/dL)^*^	162 ± 52.3
SBP (mmHg)^*^	125.7 ± 9.3
DBP (mmHg)^*^	79.0 ± 6.5
Duration of T2DM (years)^*^	7.1 ± 5.7
Comorbidity history^†^ n(%)	111 (56.6)
Hypertension, n (%)	57 (29.1)
Dyslipidemia, n (%)	36 (18.4)
Hypothyroidism, n (%)	17 (8.7)
Neuropathy peripheral, n (%)	8 (4.1)
Diabetic neuropathy, n (%)	5 (2.6)

BMI, body mass index; DBP, diastolic blood pressure; FPG, fasting plasma glucose; HbA1c, glycated hemoglobin; SBP, systolic blood pressure; T2DM, type 2 diabetes mellitus.

^*^Values presented as mean ± standard deviation.

^†^Conditions with the n ≥ 5 are presented in this table. Patients may have >1 comorbidity.

Medical histories were coded using MedDRA Version 24.1.

### Safety outcomes

**Figure 2:**
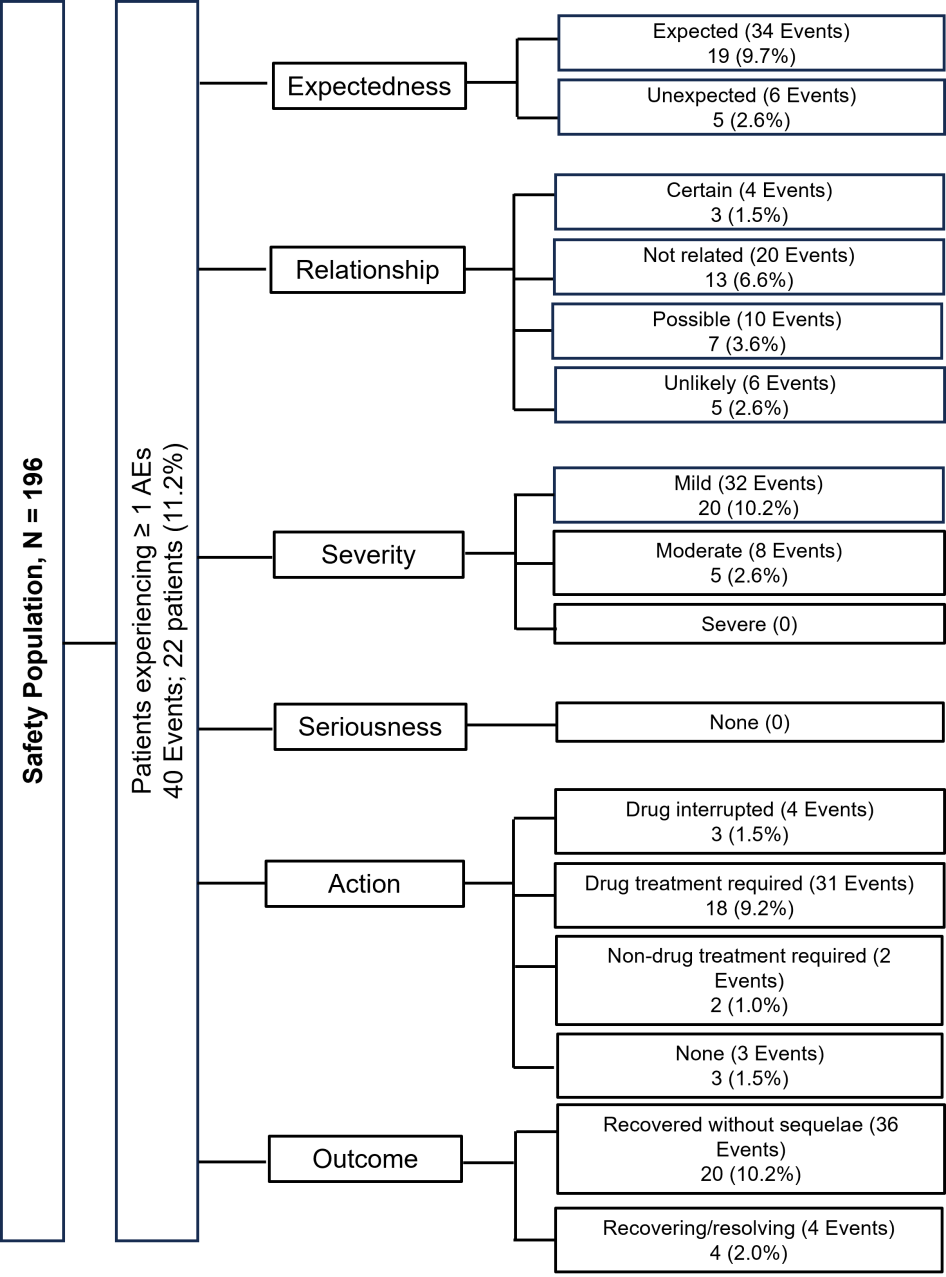
Summary of adverse events. AE, adverse event.

Of the 196 patients, data on safety were available for 142 patients (72.4%), with data missing for 54 (27.6%) patients. A total of 22 patients (11.2%) experienced 40 AEs. All AEs were treatment-emergent, and 13 (6.6%) patients had AEs that were not related to the study drug. Eighteen patients (9.2%) required drug treatment for AEs and 3 (1.5%) patients had AEs leading to temporary interruption of the study drug. Most patients (n = 20; 10.2%) had AEs that were mild in severity and recovered without sequelae and 4 (2.0%) patients were recovering at the end of follow-up. No SAEs, AEs leading to treatment discontinuation, or death were reported during the study (**Figure 2**). The most frequent AEs reported by the patients included urinary tract infection (n = 5; 2.6%), pyrexia (n = 5; 2.6%), nasopharyngitis (n = 3; 1.5%), and balanoposthitis (n = 3; 1.5%) (**Table 2**). The AESIs reported were genital tract infection (n = 3; 1.5%) and hypoglycemia (n = 1; 0.5%). The hypoglycemic event was mild and was self-treated by the patient without any treatment intervention or assistance. No new cases of HHF were reported.

**Table 2:** Treatment-emergent adverse events by system organ class and preferred term.

Safety Population (N = 196)		
AEs (≥ 1 patient)	Patients n (%)	Events (n)
	22 (11.2)	40
**Infections and infestations**	**9 (4.6)**	**13**
Urinary tract infection	5 (2.6)	7
Nasopharyngitis	3 (1.5)	3
Balanitis candida	1 (0.5)	1
Pharyngitis	1 (0.5)	1
Trichomoniasis	1 (0.5)	1
**General disorders and administration site conditions**	**5 (2.6)**	**6**
Pyrexia	5 (2.6)	5
Pain	1 (0.5)	1
**Gastrointestinal disorders**	**4 (2.0)**	**6**
Nausea	2 (1.0)	2
Vomiting	2 (1.0)	2
Abdominal pain upper	1 (0.5)	1
Gastritis	1 (0.5)	1
**Nervous system disorders**	**3 (1.5)**	**3**
Headache	1 (0.5)	1
Paraesthesia	1 (0.5)	1
Polyneuropathy	1 (0.5)	1
**Reproductive system and breast disorders**	**3 (1.5)**	**4**
Balanoposthitis	3 (1.5)	4
**Investigations**	**2 (1.0)**	**2**
Blood creatine phosphokinase increased	1 (0.5)	1
Blood glucose decreased	1 (0.5)	1
**Cardiac disorders**	**1 (0.5)**	**1**
Left ventricular dysfunction	1 (0.5)	1
**Hepatobiliary disorders**	**1 (0.5)**	**1**
Non-alcoholic fatty liver disease	1 (0.5)	1
**Immune system disorders**	**1 (0.5)**	**1**
Hypersensitivity	1 (0.5)	1
**Metabolism and nutrition disorders**	**1 (0.5)**	**1**
Hyperkalemia	1 (0.5)	1
**Musculoskeletal and connective tissue disorders**	**1 (0.5)**	**1**
Muscle spasms	1 (0.5)	1
**Respiratory, thoracic**, **and mediastinal disorders**	**1 (0.5)**	**1**
Cough	1 (0.5)	1

AEs, adverse events.

‘N’ represents the total number of patients in safety population; ‘n’ represents the total number of patients with a particular AE.Bold text indicates the System Organ Class.

No clinically significant changes were observed in hematology, clinical chemistry, urinalysis, physical examination, vital signs, or ECG during the study visits, until Week 24 (**Supplementary Tables S2, S3**).

### Efficacy outcomes

**Figure 3:**
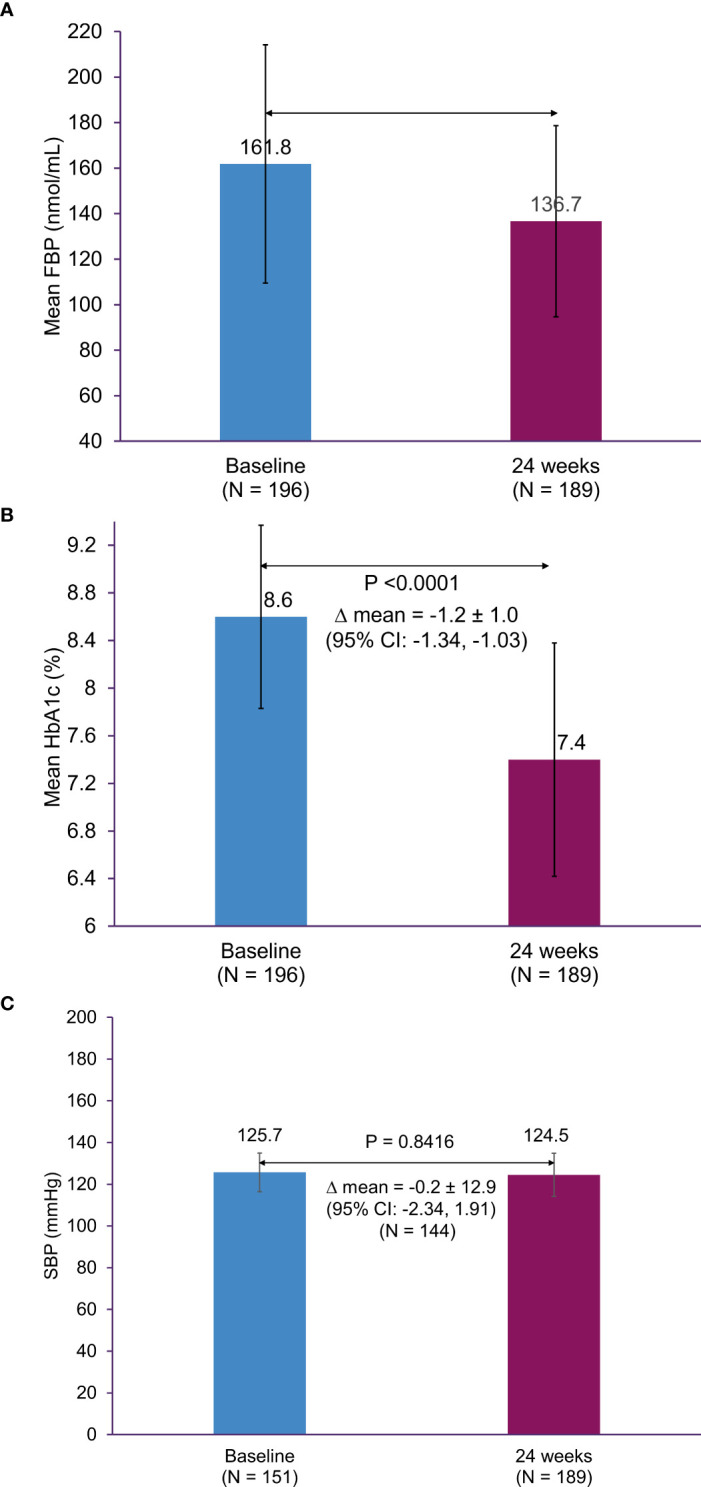
Change in HbA1c, FPG, and SBP from baseline to 24 weeks. **(A)** HbA1c, **(B)** FPG, and **(C)** SBP. CI, confidence interval; FPG, fasting plasma glucose; HbA1c, glycated hemoglobin; SBP, systolic blood pressure; SD, standard deviation.

The mean ± SD change in HbA1c from baseline to 24 weeks was -1.2% ± 1.1% (95% confidence interval [CI], -1.34 to -1.03; p < 0.0001); the mean ± SD HbA1c was 7.4% ± 1.0% at Week 24 (**Figure 3A**). The mean ± SD FPG at baseline was 161.8 ± 52.3 mg/dL, which significantly decreased to 136.7 ± 42.0 mmol/L following 24 weeks of treatment (p < 0.0001) (**Figure 3B**). Additionally, there was a significant reduction in the mean body weight at 24 weeks, compared to baseline (72.2 ± 11.5 kg versus 74.2 ± 12.6 kg, respectively); the mean ± SD change in body weight was -2.1 ± 4.0 kg (95% CI, -2.66 to -1.51; p < 0.0001). The mean ± SD change in SBP from baseline to Week 24 was -0.2 ± 12.9 mmHg (95% CI, -2.34 to 1.91; p = 0.8416) (**Figure 3C**).

Overall, the mean ± SD compliance with the study drug combination was 98.7% ± 2.9% at 8 weeks which remained consistent at 16 weeks (97.6% ± 4.6%) and 24 weeks (98.1% ± 5.9%). Overall, a median of 100% drug compliance was reported during the study.

## Discussion

The FDC of dapagliflozin and saxagliptin has the potential to improve patient outcomes among Indian patients with T2DM, who are often burdened with a high prevalence of comorbidities, thus necessitating treatment with multiple medications. Previous research has indicated that transitioning from multiple drugs to an FDC regimen led to significantly higher treatment adherence (20–23). These studies suggest that FDCs present a rational approach to T2DM management, providing enhanced treatment compliance due to reduced dosing complexity and decreased pill burden. This approach is particularly beneficial for India where several logistical and socio-economic factors may hinder access to healthcare and medication adherence. Several studies on the safety and efficacy of the FDC of dapagliflozin and saxagliptin have been conducted in regions outside of India (14, 24), but Indian patients have a distinct cardiometabolic profile. Therefore, it is essential to evaluate the safety profile of the FDC in Indian patients. A recent Indian consensus study on the use of the FDC of dapagliflozin and saxagliptin emphasizes the urgent need for its use in the Indian population (25). However, before its widespread use, it is crucial to assess its safety profile in this specific population. The present study was conducted as a part of local regulatory requirements to provide post-marketing safety data for the FDC of dapagliflozin and saxagliptin in Indian patients with T2DM. Our results indicated that the FDC was safe and well tolerated with no new safety concerns. Additionally, a significant reduction in HbA1c levels, FPG, and body weight was observed at 24 weeks.

With regards to the safety profile of the FDC of dapagliflozin and saxagliptin, there were no new unexpected safety concerns. Additionally, no new cases of HHF were reported in this study. The majority of AEs were mild in severity and were not related to the study drugs. The Phase 3 trials have reported urinary tract infections in 3.1% to 5.2% of patients, genital infections in 1.6% to 5.0%, nasopharyngitis in 3.7%, and influenza in 1.0% to 5.0%, with FDC of dapagliflozin and saxagliptin, over the 24-week treatment (11–13, 26). However, in the current study, the overall incidence of urinary tract infections was 2.6% and nasopharyngitis was 1.5%. Also, the overall incidence of nausea (1%), vomiting (1%), headache (0.5%), and cardiac disorders (0.5%) is lower than that reported in the previous studies (headache: 3.5% to 5.6% and diarrhea: 1.9% to 2.2%) (12, 13). Considering the small sample size in our study, larger studies with long-term follow-up period are needed.

A meta-analysis of 8 randomized clinical trials demonstrated that patients treated with the combination of dapagliflozin and saxagliptin experienced a higher frequency of hypoglycemia compared to either agent as an add-on to metformin. On the contrary, genital infections were less frequently reported with the combination regimen, compared to dapagliflozin alone added to metformin (14). However, in our study, AESIs were reported in 4 patients, with 1 mild hypoglycemic event (0.5%) which resolved with dietary intervention, and genital infections in 3 (1.5%) patients. These observations are consistent with a post-hoc pooled safety analysis, which revealed that the combination of dapagliflozin and saxagliptin was safe and well tolerated, whether administered concurrently or sequentially with metformin therapy, over a 24-week treatment duration (13). Moreover, there were no clinically significant abnormalities with regard to vital signs, hematology, clinical chemistry, physical examination, and ECG in our study. Overall, these findings demonstrate an acceptable safety profile of the FDC of dapagliflozin and saxagliptin as an add-on to metformin among Indian patients with T2DM.

Our study also assessed short-term glycemic control with the FDC over 24 weeks. Previous investigations have documented the mean change from baseline in HbA1c ranging from -0.5% to -1.7% at 24 weeks and from -1.2% to -1.4% over the duration of 52 weeks following treatment with the combination of dapagliflozin and saxagliptin in patients with T2DM (9–12, 27–29). Consistent with these findings, our study reported a mean change in HbA1c of -1.2% at 24 weeks. Along similar lines, prior investigations also reported a reduction in FPG levels from baseline with this combination, ranging from -9 to -38 mg/dL at 24 weeks (9–12) and -26 to -35.8 mg/dL at 52 weeks (10, 28). Our study also reports a significant mean change in FPG of -24.4 mg/dL from baseline to Week 24 (p < 0.0001), aligning with the previous observations (9–12). Further, findings from a meta-analysis indicated a significantly greater reduction in HbA1c and FPG levels from baseline with the combination of dapagliflozin and saxagliptin as add-on to metformin, compared to individual agents (14).

Weight management is a critical treatment goal for individuals with T2DM, considering the comorbidity burden in Indian patients. Accordingly, treatment guidelines advocate for the incorporation of glucose-lowering regimens that actively support patients in achieving weight reduction goals (30). Our study showed a reduction in the mean body weight (74.2 ± 12.6 kg versus 72.2 ± 11.5 kg) with the mean change from baseline of 2.1 kg following 24 weeks of treatment (p < 0.0001). These results corroborate prior findings with the mean change in body weight from baseline ranging from -1.5 to -2.1 kg at 24 weeks (9, 10, 12, 27, 29) and -2.3 to -3.2 kg at 52 weeks (10, 27, 28), with the combination of dapagliflozin and saxagliptin. These findings highlighted that effective glycemic control is achieved with the use of a combination of dapagliflozin and saxagliptin without a corresponding increase in body weight. However, further comparative studies focusing the real-world effectiveness of dapagliflozin and saxagliptin with other glucose-lowering agents may provide conclusive results.

While, recent meta-analyses have revealed a significant reduction in SBP with the dual combination of dapagliflozin and saxagliptin added to metformin, when compared to either dapagliflozin or saxagliptin added to metformin (14, 31), our study did not find a statistically significant reduction in SBP at 24 weeks.

The use of dapagliflozin and saxagliptin FDC presents a rational approach for achieving optimal glycemic control and better tolerability while minimizing pill burden. Incorporating a once-daily FDC regimen holds promise in enhancing patient compliance, particularly for Indian patients with T2DM, burdened with multiple comorbidities.

## Limitations

Limitations of this study include absence of a control group, which prevented comparison with other oral antidiabetic agents and lack of stratified efficacy assessment for HbA1c or body mass index. The short follow-up time and small sample size also limit the generalizability of our findings. Additionally, due to real-world nature of the study, safety data was missing for >25% of patients alongside. Concomitant medications including glucose-lowering medications were recorded at baseline alone, also the impact of these medications including baseline metformin on the overall safety and efficacy parameters was not analyzed. This study was conducted as a part of regulatory commitment however, with the increased utilization of the FDC, comprehensive reporting of real-world data can be generated providing further evidence on the efficacy and tolerability of the FDC in the Indian population. Conducting long-term studies with larger sample size might yield more information regarding the effectiveness and safety profile of this combination as well as the incidence of rare AEs.

## Conclusion

This was the real-world study for assessing the safety of dapagliflozin and saxagliptin FDC in Indian population. Our study demonstrated that the FDC is safe and well tolerated with no new safety signals compared to the available safety data on individual components. With significant improvement in glycemic control and body weight reduction, this FDC may be a suitable treatment option for patients with T2DM inadequately controlled with metformin. However, large-scale, long-term, comparative studies with robust methodology may help uncover further safety limitations and efficacy benefits providing greater insights into the clinical utility of this FDC.

## Data Availability

The original contributions presented in the study are included in the article/**Supplementary Material**. Further inquiries can be directed to the corresponding author.
