# Supplementary material for: Safety and efficacy of fixed-dose combination of dapagliflozin and saxagliptin in patients with type 2 diabetes mellitus – a phase 4 study in India
Source: Front Endocrinol (Lausanne). 2025 Mar 3;16:1528801. doi: 10.3389/fendo.2025.1528801 (PMC11911173; doi:10.3389/fendo.2025.1528801)
Supplement: Supplementary file 1 [file DataSheet1.docx]

**SUPPLEMENTARY TABLES AND FIGURES**

**Table S1: Methodology: eligibility criteria**

| **Inclusion criteria** | - Male and female patients aged >18 and above. - Documented history of T2DM with HbA1c level >7.0% and ≤10% at the screening visit. - Patients who were on a stable dose of anti-diabetic drugs (including metformin dose between 1000 and 2000 mg) in the past 3 months. |
| --- | --- |
| **Exclusion criteria** | - Known allergies or contraindications to the contents of the investigational product, dapagliflozin, or saxagliptin tablets. - Type 1 diabetes mellitus. - Treatment with an SGLT2i, GLP-1 agonist, or DPP4i at Visit 1 or 2. - Patients with moderate to severe renal impairment (eGFR persistently <45 mL/min/1.73 m^2^ by CKD-EPI formula or ESRD) or unstable or rapidly progressing renal disease. - Patients with severe hepatic impairment (Child-Pugh class C). - History of pancreatitis or pancreatic surgery. - Patients with a history of any malignancy. - Patients with any of the following CV/vascular diseases within 3 months prior to signing the consent at enrollment, as assessed by the investigator:   - Myocardial infarction.   - Cardiac surgery or revascularization (CABG/PTCA).   - Unstable angina.   - Transient ischemic attack or significant cerebrovascular disease.   - Unstable or previously undiagnosed arrhythmia. - History of heart failure. - Severe uncontrolled hypertension defined as SBP ≥180 mmHg and/or diastolic blood pressure ≥110 mmHg at any visit up to randomization. - History of diabetic ketoacidosis. - Any acute/chronic systemic infections. - Recurrent urogenital infections. - Patients at risk for volume depletion as judged by the investigator. - Any condition which, in the judgment of the investigator, may render the patient unable to complete the study or which may pose a significant risk to the patient or patient suspected or with confirmed poor protocol or medication compliance. |

CKD-EPI = Chronic Kidney Disease Epidemiology Collaboration; CV = cardiovascular; DPP4i = dipeptidyl peptidase 4 inhibitors; eGFR = estimated glomerular filtration rate; ESRD = end-stage renal disease; GLP-1 = glucagon-like peptide 1; HbA1c = glycated hemoglobin; SBP = systolic blood pressure; SGLT2i = sodium-glucose cotransporter-2 inhibitors; T2DM = type 2 diabetes mellitus

**Table S2: Changes in vital signs, electrocardiogram, urinalysis, and physical examination (Safety population)**

| **Parameters** | **Baseline** | **24 weeks** |
| --- | --- | --- |
| **Vital signs** | | |
| Pulse rate (beats/min)^*^ | 81.8 ± 7.6^a^ | 80.2 ± 7.2^b^ |
| Respiratory rate (breaths/min)^*^ | 18.2 ± 1.6^a^ | 17.8 ± 1.7^b^ |
| DBP (mmHg) | 79.0 ± 6.5^a^ | 77.8 ± 6.2^b^ |
| Body temperature (°F)^*^ | 97.8 ± 1.2^a^ | 97.7 ± 0.7^b^ |
| **Electrocardiogram** | | |
| Heart rate (bpm)^*^ | 82.9 ± 11.0^c^ | 80.1 ± 10.0^b^ |
| QRS (ms)^*^ | 82.3 ± 21.4^c^ | 82.3 ± 22.3^b^ |
| PR (ms)^*^ | 146.0 ± 21.1^c^ | 147.1 ± 18.7^b^ |
| RR (ms)^*^ | 746.4 ± 197.3^c^ | 760.4 ± 173.8^b^ |
| QT (ms)^*^ | 368.1 ± 33.4^c^ | 366.9 ± 28.7^b^ |
| QTcB(ms)^*^ | 419.7 ± 35.2^c^ | 416.2 ± 38.2^b^ |
| **Urinalysis N (%)** | | |
| **Blood** |  |  |
| Abnormal NCS | 6 (3.1) | 5 (2.8) |
| Normal | 190 (96.9) | 173 (97.2) |
| **Protein** |  |  |
| Abnormal NCS | 56 (28.6) | 24 (13.5) |
| Normal | 140 (71.4) | 154 (86.5) |
| **Glucose** |  |  |
| Abnormal NCS | 83 (42.3) | 86 (48.3) |
| Normal | 112 (57.1) | 92 (51.7) |
| **Physical Examination N (%)** | | |
| **Lungs** |  |  |
| Abnormal NCS | 0 | 0 |
| Normal | 185 (100) | 177 (99.4) |
| **Neurological System** |  |  |
| Abnormal NCS | 0 | 0 |
| Normal | 185 (100) | 177 (99.4) |
| **Dermatological System** |  |  |
| Abnormal NCS | 0 | 0 |
| Normal | 185 (100) | 177 (99.4) |
| **Cardiovascular System** |  |  |
| Abnormal NCS | 0 | 0 |
| Normal | 185 (100) | 177 (99.4) |
| **Genito-urinary System** |  |  |
| Abnormal NCS | 0 | 1 (0.6) |
| Normal | 185 (100) | 176 (98.9) |
| **Abdomen** |  |  |
| Abnormal NCS | 0 | 0 |
| Normal | 185 (100) | 177 (99.4) |
| **General Appearance** |  |  |
| Abnormal NCS | 0 | 0 |
| Normal | 185 (100) | 177 (99.4) |
| **Extremities** |  |  |
| Abnormal NCS | 1 (0.5) | 0 |
| Normal | 184 (99.5) | 177 (99.4) |
| **Head Eye Ear Nose Throat** |  |  |
| Abnormal NCS | 0 | 0 |
| Normal | 185 (100) | 177 (99.4) |

DBP = diastolic blood pressure; NCS = non-clinically significant

*Data presented as mean ± SD.

Percentages are calculated based on the number of patients with data available.

^a^N = 151; ^b^N = 178; ^c^N = 192

**Table S3: Clinical chemistry and hematology parameters (Safety population)**

| **Parameters** | **Observed Value**  **(Mean ± SD)** | **Observed Value (Mean ± SD)** | **Change from Baseline**  **(Mean ± SD)** |
| --- | --- | --- | --- |
|  | **Baseline** | **24 weeks** | |
| **Clinical Chemistry** | | | |
| Creatinine (mg/dL) | 0.8 ± 0.2^a^ | 0.8 ± 0.2^b^ | 0.0 ± 0.2^b^ |
| Total Bilirubin (mg/dL) | 0.6 ± 0.3^a^ | 0.6 ± 0.3^b^ | -0.0 ± 0.3^b^ |
| Alkaline phosphatase (U/L) | 92.7 ± 26.9^a^ | 92.0 ± 21.8^b^ | -1.2 ± 30.7^b^ |
| Aspartate transaminase (U/L) | 26.2 ± 10.8^a^ | 24.9 ± 7.9^b^ | -1.6 ± 11.4^b^ |
| Alanine transaminase (U/L) | 30.8 ± 17.7^a^ | 27.2 ± 12.6^b^ | -4.0 ± 17.5^b^ |
| Albumin (g/dL) | 4.4 ± 0.4^a^ | 4.4 ± 0.4^b^ | -0.1 ± 0.6^b^ |
| Potassium (mEq/L) | 4.4 ± 0.6^a^ | 4.5 ± 0.5^b^ | 0.1 ± 0.6^b^ |
| Total Calcium (mg/dL) | 9.5 ± 0.6^a^ | 9.5 ± 0.5^c^ | -0.0 ± 0.7^c^ |
| Sodium (mmol/L) | 137.6 ± 12.0^d^ | 139.4 ± 2.8^e^ | 0.7 ± 3.7^e^ |
| Sodium (mEq/L) | 139.3 ± 3.7^f^ | 139.5 ± 3.8^g^ | 0.2 ± 5.0^g^ |
| Creatine kinase (U/L) | 78.7 ± 53.8^h^ | 89.6 ± 53.9^i^ | 11.2 ± 56.8^j^ |
| **Hematology** | | | |
| Hematocrit (%) | 41.1 ± 5.1^a^ | 42.6 ± 4.29^b^ | 1.5 ± 5.15^b^ |
| Leukocyte count (/μL) | 8360.2± 6151.7^a^ | 8093.2 ± 7441.6^b^ | -356.6 ± 9854.0^b^ |
| Neutrophils (%) | 60.6 ± 7.7^a^ | 60.8 ± 7.4^b^ | 0.3 ± 9.7^b^ |
| Basophils (%) | 0.1 ± 0.2^a^ | 0.1 ± 0.2^b^ | -0.0 ± 0.1^b^ |
| Lymphocytes (%) | 30.4± 7.3^a^ | 30.5 ± 6.8^b^ | -0.1 ± 8.8^b^ |
| Eosinophils (%) | 3.2 ± 2.4^a^ | 2.9 ± 1.6^b^ | -0.3 ± 2.4^b^ |
| Monocytes (%) | 5.7 ± 2.1^a^ | 5.6 ± 2.5^b^ | -0.1 ± 2.4^b^ |
| Platelet count (/μL) | 226379.8 ± 83159.8^a^ | 245214.2 ± 71253.2^b^ | 16573.6 ± 80320.4^b^ |
| Hemoglobin (g/dL) | 13.4 ± 1.8^a^ | 13.7 ± 1.5^b^ | 0.3 ± 1.6^b^ |

SD = standard deviation

^a^N = 196; ^b^ N = 178; ^c^N = 177; ^d^N = 134; ^e^N = 118; ^f^N = 61; ^g^N = 59; ^h^N = 184; ^i^N = 170; ^j^N = 163

**Supplementary Figure S1: Study design**

AE = adverse event; ECG = electrocardiogram; EOS = end of study; EOT = end of treatment; FPG = fasting plasma glucose; HbA1c = glycated hemoglobin; TC = telephonic consultation

Enrollment and Visit 2 can coincide to enter the patient into the study.

At Visits 3, 4, 6, and 8, assessment of AEs was performed telephonically.

ECG was measured at Visits 2, 7, and 9.
