# Supplementary figures and images for: Safety and efficacy of fixed-dose combination of dapagliflozin and saxagliptin in patients with type 2 diabetes mellitus – a phase 4 study in India
Source: Front Endocrinol (Lausanne). 2025 Mar 3;16:1528801. doi: 10.3389/fendo.2025.1528801 (PMC11911173; doi:10.3389/fendo.2025.1528801)

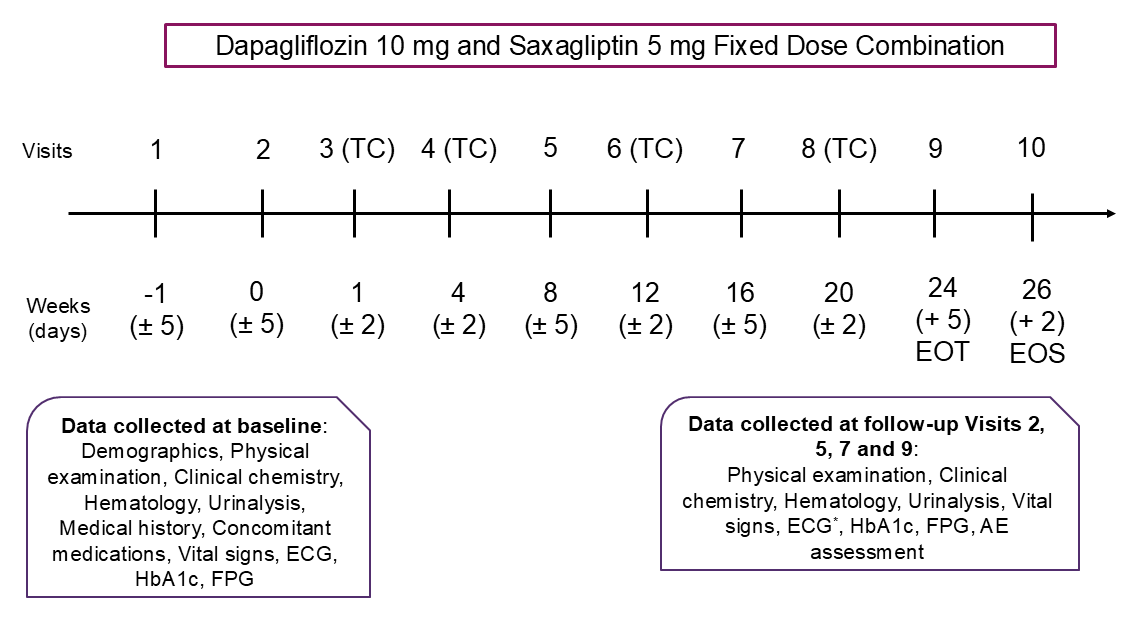

Supplement: Supplementary file 2 [file Image1.tif]
